# Concord and Niagara Grape Juice and Their Phenolics Modify Intestinal Glucose Transport in a Coupled in Vitro Digestion/Caco-2 Human Intestinal Model

**DOI:** 10.3390/nu8070414

**Published:** 2016-07-05

**Authors:** Sydney Moser, Jongbin Lim, Mohammad Chegeni, JoLynne D. Wightman, Bruce R. Hamaker, Mario G. Ferruzzi

**Affiliations:** 1 Department of Food Science, Purdue University, West Lafayette, IN 47907, USA; moser6@purdue.edu (S.M.); lim175@purdue.edu (J.L.); mchegeni@purdue.edu (M.C.); mferruzz@purdue.edu (M.G.F.); 2 Welch Foods Inc., Concord, MA 01742, USA; jwightman@welchs.com; 3 Whistler Center for Carbohydrate Research, Purdue University, West Lafayette, IN 47907, USA

**Keywords:** grape juice, anthocyanins, carbohydrate digestion, glucose transport

## Abstract

While the potential of dietary phenolics to mitigate glycemic response has been proposed, the translation of these effects to phenolic rich foods such as 100% grape juice (GJ) remains unclear. Initial in vitro screening of GJ phenolic extracts from American grape varieties (V. labrusca; Niagara and Concord) suggested limited inhibitory capacity for amylase and α-glucosidase (6.2%–11.5% inhibition; *p* < 0.05). Separately, all GJ extracts (10–100 µM total phenolics) did reduce intestinal trans-epithelial transport of deuterated glucose (d7-glu) and fructose (d7-fru) by Caco-2 monolayers in a dose-dependent fashion, with 60 min d7-glu/d7-fru transport reduced 10%–38% by GJ extracts compared to control. To expand on these findings by assessing the ability of 100% GJ to modify starch digestion and glucose transport from a model starch-rich meal, 100% Niagara and Concord GJ samples were combined with a starch rich model meal (1:1 and 1:2 wt:wt) and glucose release and transport were assessed in a coupled in vitro digestion/Caco-2 cell model. Digestive release of glucose from the starch model meal was decreased when digested in the presence of GJs (5.9%–15% relative to sugar matched control). Furthermore, transport of d7-glu was reduced 10%–38% by digesta containing bioaccessible phenolics from Concord and Niagara GJ compared to control. These data suggest that phenolics present in 100% GJ may alter absorption of monosaccharides naturally present in 100% GJ and may potentially alter glycemic response if consumed with a starch rich meal.

## 1. Introduction

On average, Americans consume 0.43 gallons per capita of grape juice (GJ) annually, making GJ the third most commonly consumed juice in the US [1]. Native American Concord and Niagara grape cultivars are sources for production of purple and white juice, respectively. Both grapes and their corresponding juices are well established sources of nutrients and bioactive phenolic compounds, including flavan-3-ols, flavonols, stilbenes, phenolic acids and, for Concord grapes, anthocyanins [2,3,4,5]. With total phenolic and anthocyanin levels reported as high as 2900 mg/L and 880 mg/L, respectively, for 100% Concord GJ and similarly high levels of phenolics in 100% Niagara GJ, these products can be significant contributors to health promoting phytochemicals [4]. 

Phenolic rich 100% Concord GJ consumption has been reported to have health promoting activities including improved cardiovascular and cognitive function [6,7,8]. Primary outcomes mediated by Concord GJ include increased flow mediated dilation, decreased platelet aggregation, modulation of low density lipoprotein (LDL) oxidation lag time, and improved memory function and brain signaling (reviewed by Blumberg et al. [6]; Krikorian et al. [7,8]). While promising, these benefits have been observed with consumption of between ~12 and 20 oz of 100% juice and 100% GJ contains ~36 g sugar per 240 mL serving [9]. While fruit juices have been reported to have similar glycemic response to whole fruits when matched by sugar load [10], realization of these benefits remains challenged by consumer concern related to the higher sugar content and risk for obesity and diabetes. Therefore, while evidence for the benefits of GJ and GJ phenolics continues to expand, there remains a hesitation in recommending 100% GJ consumption to certain populations due to its natural high sugar content. 

Over the past decade, the potential of phenolics to modulate glucose homeostasis has emerged (reviewed by Hanhineva et al. [11] and Williamson et al. [12]). Specifically, phenolics derived from foods including berries, juices, tea and coffee have demonstrated the ability to modulate intestinal digestion of starch by inhibition of amylase and glucosidase enzymes as well as intestinal glucose absorption through inhibition of glucose transporters such as GLUT 2 [13,14,15,16,17,18,19,20]. While primarily based on experiments with purified phenolics and phenolic extracts, these data would suggest that sugar in the context of a phenolic rich food or beverage may be processed differently in the intestine resulting in a modified glycemic response. Interestingly, while similar glycemic responses have been observed between grape juice and fruit, juice resulted in lower insulin response [21]. Johnston et al. [22] previously reported that 3 h glycemic response from both clear and cloudy 100% apple juice was in fact lower in healthy volunteers compared to a phenolic-free, sugar-matched placebo beverage. While no mechanistic test were performed, the authors postulated that phenolics in apple juices, including phloridzin or other polyphenols, may be responsible, in part, for the observed delay in intestinal absorption of glucose. In a related fashion, chronic consumption of 100% Concord GJ (8 weeks) decreased fasting blood glucose levels compared to placebo beverage [23]. These results do in fact suggest that specific 100% juice components might modify glucose absorption and/or homeostasis in humans. 

While these data are promising, additional insight into the ability and mechanisms by which GJ phenolics may modulate glucose absorption is required. Also, considering the potential mechanism of phenolic inhibition of starch digestive enzyme and glucose transport, it is important to consider the potential impact of a complex meal on these effects. Although clinical studies remain the gold standard for investigating health-related outcomes, in vitro models provide an effective screening tool to investigate mechanistic steps and screen a broader set of food matrix factors that impact nutrient and phytonutrient bioavailability prior to refining designs for clinical evaluation [24,25,26,27,28]. Leveraging a similar approach, the specific objectives of this study were to (1) examine the potential for 100% GJ phenolics to modulate carbohydrate digestion and intestinal glucose transport in vitro; and (2) determine if bioaccessible phenolics from 100% GJ can alter carbohydrate digestion and intestinal glucose transport in the presence of a starch rich meal using a coupled in vitro digestion/Caco-2 model.

## 2. Materials and Methods 

### 2.1. Chemicals, Solutions and Standards

Chromatography solvents, acids and salts including acetonitrile, methanol and water, formic acid and ammonium formate in addition to phenolic standards (gallic acid, caffeic acid, epicatechin, quercetin-3-*O*-glucoside, quercetin-3-glucuronide, quercetin, resveratrol, and cyanidin-3-*O*-glucoside) were purchased from Sigma-Aldrich, (St. Louis, MO, USA). Material for test meal including nonfat dry milk (NFDM; Maple Island, North St. Paul, MN, USA) and corn starch (Tate and Lyle) were purchased at a local market. Materials for in vitro digestion including urea (U5378), uric acid (U2625), porcine mucin (M2378), α-amylase (A3176), pepsin (P7125), lipase (L3126), pancreatin (P1750) bile salt (B8631) extract, KH_2_PO_4_ (VWR), K_2_SO_4_ (Riedel-de Haën), potassium citrate, sodium citrate, KCl, CaCO_3_, MgCO_3_ (Sigma-Aldrich) and Tris-HCl were also purchased from Sigma-Aldrich. NaCl, HCl, and NaHCO_3_ were purchased from Mallinckrodt (Phillipsburg, NJ, USA). Reagents for enzyme inhibition assays including dimethylsulfoxide (DMSO), acarbose, phosphoric acid, α-amylase (A3176), rat intestinal α-glucosidase, NaCl, glucose oxidase-peroxidase, maltose, maltotriose, maltotetrose and maltopentose, were obtained from Sigma-Aldrich. Cell culture reagents including Dulbecco’s Modified Eagles Medium (DMEM), non-essential amino acids (NEAA), penicillin/streptomycin (pen/strep), and phosphate buffered saline (PBS) were purchased from Lonza (Walkersville, MD, USA). Cell culture reagents including 4-(2-hydroxylethyl)-1-piperazineethanes (HEPES), bovine serum albumin (free fatty acid free) (FFA) and glucose-free DMEM were purchased from Sigma-Aldrich. NaHCO_3_, monosodium phosphate, and disodium phosphate were obtained from J.T. Baker (Center Valley, PA, USA). Fetal bovine serum (FBS) (Atlanta Biologicals, Lawrenceville, GA, USA), gentamycin (J.R. Scientific, Woodland, CA, USA), trypsin (Thermo Scientific, Waltham, MA, USA), glucose and fructose (Research Products International Corps, Mt. Prospect, IL, USA), and d-glucose-1,2,3,4,5,6,6-d7 (d7-glu) and d-Fructose-1,1,3,4,5,6,6-d7 (d7-fru) (Sigma-Aldrich) were used in glucose transport experiments. 

### 2.2. Grape Juice Samples

One hundred percent Niagara and Concord GJ were provided by Welch Foods Inc. (Concord, MA, USA) (Table 1). One hundred percent juices were produced from two harvest years (2013 and 2014) and were pasteurized and maintained refrigerated at 4 °C until used in experiments. The Niagara GJs were processed with and without the addition of sulfur dioxide. 

### 2.3. Phenolic Extraction 

Phenolics were extracted from aliquots (5 mL) of GJ by solid phase extraction (Oasis^®^ HLB 6cc (150 mg) extraction cartridges) using the method of Song et al. [29]. Briefly, 5 mL of juice were loaded onto the SPE cartridges (Milford, MA, USA) and rinsed with 2% formic acid in water. Elution of phenolics was completed with 2% formic acid in methanol. Eluates were dried down under nitrogen and kept frozen (−80 °C) until analysis. Total phenolic content of extracts was measured using a modified Folin-Ciocalteau assay as described by Waterhouse et al. [30]. 

### 2.4. Analysis of Polyphenol and Anthocyanin-Rich Fractions by LC-MS 

Dried GJ extracts were resolubilized in 2.0% formic acid in water and characterized by LC-MS using methods of Song et al. [29] with minor modification. Single ion responses (SIRs) were used to quantify individual GJ phenolics. Phenolic acids, flavonoids and stilbenoids were quantified using multi-level response curves constructed with authentic standards of each phenolic species identified by co-chromatography with limited exception. Piceid concentration (a resveratrol glucoside) was estimated using a resveratrol calibration curve. Concentration of all quercetin-*O*-glucosides was estimated using quercetin-3-*O*-glucoside. Finally, concentrations of anthocyanins were determined using a calibration curve constructed from cyanidin-3-*O*-glucoside.

### 2.5. Impact of GJ Phenolic Extracts on Starch Digestive Enzymes in Vitro

Impact of GJ phenolic extracts on starch digestion by α-amylase and α-glucosidase was determined as described by Lee et al. [31]. Briefly, GJ phenolic extracts were dissolved in DMSO (5 mM). A waxy maize starch solution (1 g/50 mL) was prepared in 20 mM sodium phosphate buffer and boiled to achieve gelatinization (20 min). Starch solutions (50 µL) were then combined with GJ phenolic extract (15 µL, delivering 10–500 μM of total phenols), pancreatic α-amylase (37 °C, 10 U/37.5 µL) and phosphate buffer (20 mM, pH 6.8). Samples were incubated for 10 min after which the reaction was terminated by boiling. Samples were diluted 10× in water prior to quantification of maltose and maltotriose by HPAEC-ECD. Percent inhibition of α-amylase by GJ phenolics was calculated relative to vehicle control (DMSO with no GJ extract) and compared to positive control acarbose.

Inhibition of α-glucosidase by GJ phenolics was assessed using a Megazyme glucose assay kit (Megazyme Inc., Chicago, IL, USA). Briefly, rat intestinal α-glucosidase solution (1 g/10 mL, 10 µL) was mixed with inhibitor (10 µL, 100–5000 µM total polyphenols). Sodium phosphate (0.1 M, pH 6.8, 70 µL) was added to the enzyme-inhibitor solution and the mixture was vortexed well. Maltose solution (10 µL, 100 mg/mL) was then added. The reaction mixture was incubated at 37 °C for 90 min. Enzymes were inactivated by placing the samples in boiling water. Samples were then centrifuged. The supernatant was collected and diluted 10×. Glucose content was determined by addition of glucose oxidase-peroxidase reagent and measuring absorbance at 510 nm. Percent inhibition of α-glucosidase by phenolic extracts and acarbose was determined by comparing the difference in absorbance between control and extract relative to absorbance of the control.

### 2.6. Inhibition of Glucose/Fructose Transport through Caco-2 Human Intestinal Cell Monolayers by GJ Phenolic Extracts

Potential for inhibition of glucose and fructose intestinal transport by GJ phenolic extract was assessed using a three-compartment Caco-2 human intestinal cell culture model. Caco-2 (TC7 clone) cells were maintained in DMEM supplemented with 10% *v*/*v* FBS, 1% *v*/*v* NEAA, 1% *v*/*v* HEPES, 1% *v*/*v* pen/strep and 0.1% *v*/*v* gentamicin. Cells were seeded (2.12 × 10^5^ cells/cm^2^), grown and differentiated in 6 well insert plates (Corning^®^ Transwell^®^ polyester membrane, Corning Inc., Oneonta, AL, USA, 24 mm diameter, pore size 0.4 μm) under a humidified atmosphere of air/CO_2_ (95:5) at 37 °C. All experiments used differentiated monolayers (electrical resistance >250 Ω) at passages 90–95, with transport studies conducted 21–24 days post-confluency. Cells were incubated in glucose-free DMEM for 2 h preceding treatment. Test media for initial experiments was prepared by solubilizing glucose and fructose (9 mM each), d7-glu and d7-fru (3 mM each), and GJ phenolic extracts in PBS pH 5.5 (delivering 10–100 µM total phenolics, respectively). Cellular viability was assessed using the MTT assay (Biotium, Hayward, CA, USA). Highly differentiated cell monolayers treated with phenolic extracts and digesta (at concentrations >100 µM) for 4 h were found to have >95% viability. Test media was applied to the apical surface of cell monolayers. After 60 min incubation, basolateral and apical media were collected and cells were washed twice with 0.1% fatty acid free PBS. Membranes were then washed with ice cold PBS to terminate glucose transport, and cells were collected by scraping and frozen until analysis. All treatments were performed in quadruplicate.

### 2.7. Analysis of d7-Glucose and d7-Fructose Concentration in Basolateral Media by LC-MS

Basolateral media (100 µL) was extracted using acetone (0.5 mL), dried down under nitrogen, resolubilized in mobile phase (0.6 mL), and centrifuged (14,000 rpm, 5 min) prior to analysis for the chlorine adduct of d7-glu and d7-fru by LC-TOF-MS [32]. 10 μL of sample was injected on a Waters ACQUITY UPLC H-Class system equipped with an ACQUITY QDa mass detector. Separation was achieved according to a method by Liu et al. [33] with minor modification. A Waters BEH-amide column (2.1 mm id × 150 mm, 2.5 μM particle size) was heated to 30 °C under isocratic conditions with flow rate of 0.65 mL/min for 6 min and mobile phase 87:13 acetonitrile:water with 8 mM ammonium formate, pH 9.8. Conditions were as follows: ionization mode: ESI (−); mass: 222 *m*/*z*; capillary voltage: 0.8 kV; cone voltage: 20 V; probe temp: 350 °C; desolvation temp: 600 °C. Glucose, fructose, d7-glu and d7-fru concentrations were calculated using calibration curves made from authentic standards.

### 2.8. Impact of 100% GJ on Glucose Release/Transport in a Coupled in Vitro Digestion/Caco-2 Model 

To extend beyond GJ extracts, a coupled three-stage in vitro digestion/Caco-2 model was used to determine if bioaccessible GJ phenolics could inhibit starch digestion and/or glucose transport with or without a co-consumed starch rich meal. Initially 100% GJ or sugar matched phenolic free control (~10 mL) was introduced to a three-stage in vitro digestion with oral, gastric and small intestinal phase as described by Moser et al. [34] and modified according to conditions described by Vermeirssen et al. [35] to include rat intestinal powder (0.15 g/reaction) as a source of α-glucosidase. Aliquots of undigested beverage starting material (SM), and centrifuged aqueous intestinal digesta (AQ) containing bioaccessible GJ phenolics were collected and acidified with aqueous acetic acid (1% total in sample) and stored frozen at −80 °C until phenolic analysis by HPLC-MS (outlined above). A separate aliquot of AQ digesta was then diluted 2:7 with PBS (pH 5.5) (delivering ~21–56 µM total bioaccessible GJ phenolics, ~24 mM monosaccharides), spiked with d7-glu (6 mM), and applied to the apical surface of Caco-2 monolayers. A matching phenolic-free control was prepared by solubilizing glucose and fructose (24 mM total) with d7-glu (6 mM each) in blank AQ digesta diluted 2:7 with PBS. Treatments were replicated in quadruplicate. Transport of d7-glu was followed as described previously. 

In a second experiment, 100% GJ was co-digested with a test meal consisting of a starch/nonfat dry milk model meal. The model meal was prepared by mixing corn starch and non-fat dry milk (NFDM) in double-distilled water (10% *v*/*v* each). The mixture was then heated (95 °C, 30 min) and cooled slowly to 4 °C. The resulting product was blended and an aliquot (5 g) was combined with 2.5 or 5 g of 100% GJ (Concord or SO_2_ Niagara, 2013 harvest) or sugar-matched control beverage (50:50 Glucose:Fructose; 16° Brix) prior to introduction to the oral phase of digestion. Starting materials (GJ plus model meal) and final AQ digesta were collected and stored (−80 °C). Bioaccessibility of phenolics was determined by comparing individual phenolic content of AQ digesta relative to starting material. The extent of starch digestion was determined by comparison of initial glucose content in starting material to that in final AQ digesta. Percentage inhibition of starch digestion by GJ was determined by comparing release of glucose from starch during digestion of model meal with grape juice relative to phenolic-free control. Following digestion, the ability of co-digested GJ to further inhibit glucose transport was determined by diluting AQ digesta 2:7 with PBS (pH 5.5) containing 6 mM d7-glu (delivering ~5–16 µM total bioaccessible phenolics, determined using Folin-Ciocalteu assay [30]) and applying to the apical surface of Caco-2 monolayers. Feeding material containing AQ from high and low level GJ samples contained ~12 mM and 6 mM glucose and fructose. Matching phenolic-free controls were prepared. Treatments were replicated in quadruplicate. Transport of d7-glu (6 mM) was tracked and compared to control matched for sugar content.

### 2.9. Data Analysis

Data for polyphenol, anthocyanin, and (d7)-glu and (d7)-fru content of GJ samples, AQ digesta and basolateral material are expressed as mean ± SEM. Relative (%) bioaccessibility is defined as the percentage of polyphenol recovered in final digesta from that in starting material. Absolute bioaccessibility (μM) is the amount of phenolic available in digesta relative to that present in starting material, calculated by multiplying % bioaccessibility by concentration (μM) of phenolic in starting material. Percent (%) glucose release from corn starch by α-amylase was calculated as the fraction of glucose released compared to negative control. Glucose transport is expressed as concentration (μM) of d7-gluc appearing in the basolateral compartment over time. Percent (%) glucose transport was calculated on the basis of initial d7-glu content in the apical compartment at time 0. In order to facilitate comparison between treatments and control for variability between individual replicates, percentage (%) glucose transport was normalized using the d7-glu transport from control. Differences in phenolic profile, bioaccessibility data, enzyme inhibitory activity and glucose transport for each GJ or GJ extract were performed using JMP (Version 12, SAS Institute, Cary, NC, USA), and evaluated using Tukey’s test or *t*-test. All significant differences testing used α < 0.05. 

## 3. Results

### 3.1. Phenolic and Anthocyanin Profiles of 100% Grape Juice

Phenolic content including anthocyanins and non anthocyaninin phenolics (Table 2) in Niagara and Concord GJ were comparable to that reported previously [5,36]. Several phenolic species previously reported in these native American grape varieties were observed including phenolic acids, flavonoids, stilbenes and anthocyanins. Concord GJ had higher levels of total phenolics compared to Niagara GJ for both 2013 and 2014 harvest juices. Further, GJ produced from 2013 harvest grapes had higher (*p* < 0.05) levels of caftaric acid, epicatechin, quercetin, quercetin-3-*O*-glucoside and specific anthocyanins compared to that from 2013 harvest grapes. Overall, phenolic acids, quercetin, and resveratrol were the most prominent phenolics observed in all samples, with levels up to 1134 µM. Use of SO_2_ during Niagara GJ processing did result in higher phenolics levels in finished juice compared to untreated juice (*p* < 0.05). 

Anthocyanins were present in GJ at lower levels compared to other phenolics (Table 2). Consistent with a report by Wang et al. [37], cyanidin and delphinidin derivatives were primary contributors to total anthocyanin content in Concord GJs. Concord GJ contained 2427.5–3092.0 ng/100 mL total anthocyanins compared to 111.1–131.9 mg/100 mL non-anthocyanin phenolics. Specifically, cyanidin-3,5-*O*-diglucoside and delphinidin-3-*O*-glucoside were the most abundant anthocyanins in Concord GJ, present up to 710.0 and 877.4 ng/100 mL, respectively. Further, cyanidin-3-*O*-p-coumaroyl-5-*O*-diglucoside and delphinidin-3-*O*-p-coumaroylglucoside were the only anthocyanins detected and tentatively identified in Niagara GJ and only at low levels (25.8–55.0 and 111.0–124.4 nmol/100 mL, respectively). 

### 3.2. Modulation of α-Amylase and α-Glucosidase Activity by Grape Juice Phenolic Extracts

The ability of GJ phenolic extracts (50–500 μM) to inhibit α-amylase and α-glucosidase was determined in vitro. Only results from higher level phenolics experiments (300 and 500 µM) are shown (Table 3) as no activity was observed at less than 300 µM (data not shown). For both assays, the positive control (acarbose) decreased α-amylase and α-glucosidase activity significantly (*p* < 0.05) with complete inhibition of α-amylase activity observed at 300 µM. α-Glucosidase inhibition was observed by 300 µM and 500 µM acarbose at 88.9% and 92.4%, respectively.

GJ phenolic extracts demonstrated only modest inhibitory capacity for α-amylase and α-glucosidase (Table 3). At 500 µM, GJ phenolic extracts only modestly decreased α-amylase activity compared to phenolic-free control, with 2013 extracts (7.9%–9.4% inhibition) generally having greater (*p* < 0.05) impact compared to 2014 extracts (0.7%–9.2% inhibition). α-Amylase inhibition at lower GJ phenolic levels was not observed. Conversely, all GJ extracts exhibited modest α-glucosidase inhibitory capacity at both 300 and 500 µM. The 2013 harvest GJ extracts had similar inhibitory capacity for α-glucosidase (5.4%–11.5% inhibition) compared to 2014 extracts (3.8%–9.3% inhibition). 

### 3.3. Modulation of Glucose Transport across Caco-2 Intestinal Cells by Grape Juice Phenolic Extracts 

**Figure 1:**
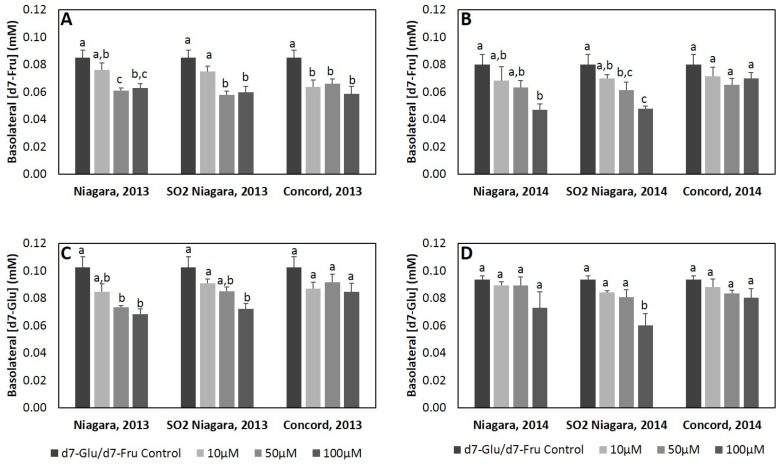
Impact of 2013 and 2014 harvest grape juice extracts on d7-fructose (**A**,**B**) or d7-glucose (**C**,**D**) transport across Caco-2 human intestinal cell monolayer. Data is represented as concentration of deuterated sugar in basolateral compartment at 60 min. Data represent mean ± SEM for *n* = 4 replicate wells at each time point. Presence of different letters between values indicates significant differences in glucose transport between treatments within each concentration (*p* < 0.05).

Previous studies have shown that various plant-derived phenolic extracts have ability to decrease basolateral glucose transport [18,19,20]. To determine if GJ phenolics exhibit similar activity, their ability to modulate intestinal glucose and fructose transport was assessed using a three-compartment Caco-2 human intestinal cell model. GJ extracts between 10 and 100 µM of total phenolics were able to reduce transport of d7-fru and d7-glu compared to control, with effect generally being increased with increased concentration (Figure 1; Table S1). Inhibition was similar across all GJ extracts and was greater for d7-fru compared to d7-glu transport, ranging from 10.9% to 41.3% and from 4.7% to 35.7% inhibition for d7-fru and d7-glu transport, respectively. Overall, extracts from Niagara GJ with and without SO_2_ had similar effect on transport of d7-glu and d7-fru and generally exhibited a greater ability to inhibit transport relative to Concord GJ extracts.

### 3.4. Influence of Bioaccessible Phenolics from 100% Grape Juice Phenolics on Carbohydrate Digestion and Glucose Transport When Co-Digested with Starch Rich Model Test Meal 

**Figure 2:**
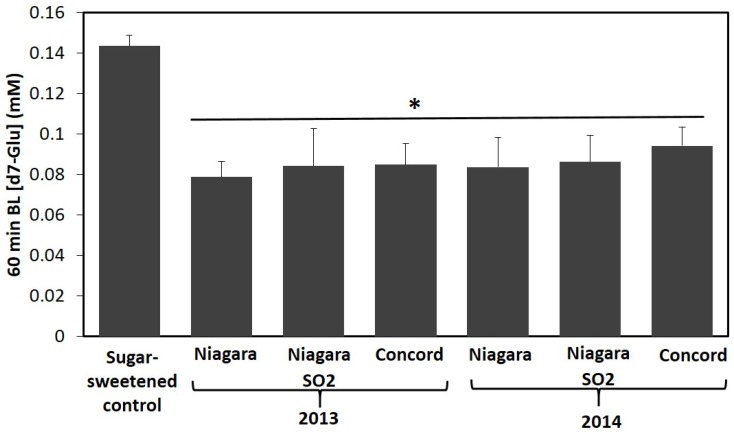
Impact of 2013 and 2014 100% grape juice aqueous digesta (AQ) on d7-glucose transport across Caco-2 human intestinal cell monolayers over 60 min. Data represent mean ± SEM for *n* = 4 replicate wells. * indicates significant difference in basolateral glucose concentration (mM) compared to control (*p* < 0.05).

**Figure 3:**
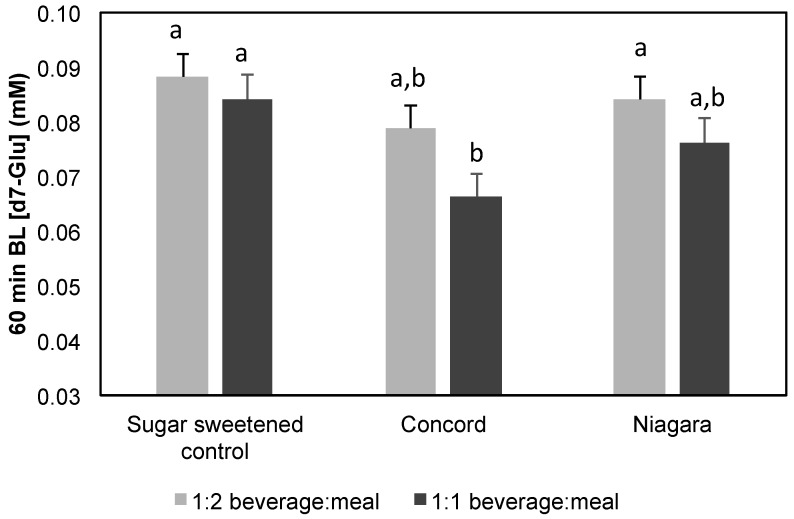
d7-Glucose transport across Caco-2 human intestinal cell monolayers from AQ digesta of co-digested GJ and starch rich test meal. Data is represented as a concentration of deuterated glucose in basolateral compartment at 60 min by treatment compared to control over 60 min. Data represent mean ± SEM for *n* = 4 replicate wells at each time point. Presence of different letter between values indicates significant differences in d7-glucose transport between treatments within the same ratio of beverage to meal.

In order to better understand the extent to which inhibition of starch digestion and glucose transport from GJ extracts translates to 100% GJ and whole food systems, Niagara and Concord GJs were subjected to an in vitro gastrointestinal digestion model with and without a starch based model meal. Bioaccessibility of GJ phenolics from each juice was determined to evaluate differences in delivery of phenolics in the upper GI tract from AQ digesta based on grape variety (Concord, Niagara), harvest year (2013, 2014), and SO_2_ treatment (Table 4). The impact of bioaccessible phenolics on intestinal transport of glucose was also assessed (Figure 2). Additionally, the impact of co-digestion of GJ with a starch rich model meal on bioaccessibility of phenolics from GJ, starch digestion, and intestinal transport of glucose was assessed (Table 5 and Table 6; Figure 3 and Table S2).

Overall, relative bioaccessibility (Table 4) of non-anthocyanin phenolics was similar between Concord and Niagara GJ varieties and generally consistent with previous reports of phenolic bioaccessibility from fruit juices [38,39,40]. SO_2_ treatment generally did not significantly alter bioaccessibility of non-anthocyanin phenolics from Niagara GJ. Phenolic acids were the most bioaccessible forms in GJ ranging from 22.0% to 56.0% relative bioaccessibility. Overall, caftaric acid was the most bioaccessible non-anthocyanin phenolic from GJ, with bioaccessibility ranging from 30.3% to 56.0%. Remaining phenolic acids as well as epicatechin and resveratrol were less bioaccessible (~11.8% to 44.7%). Quercetin, isorhamnetin, and piceid generally had higher (*p* < 0.05) relative bioaccessibility from 2013 harvest Niagara GJ compared to 2014 harvest. Although there were few trends for differences in relative bioaccessibility of phenolic acids between GJs, absolute bioaccessibility (µM) of most non-anthocyanin phenolics were significantly (*p* < 0.05) higher from SO_2_ Niagara compared to non SO_2_ treated Niagara GJ and phenolic acids and epicatechin were significantly higher (*p* < 0.05) from Concord GJ compared to Niagara GJs (Table 4).

Relative bioaccessibilities of individual anthocyanins from GJ were generally lower compared to other phenolics, ranging from 3.7% to 37.2% (Table 4). Notably, malvidin-3-*O*-glucoside had the highest bioaccessibility among anthocyanins (37.2% ± 1.7%) from Concord 2013 harvest GJ. Similar to the trend for phenolics, relative bioaccessibility for anthocyanins were higher (*p* < 0.05) from 2013 harvest compared to 2014 harvest Concord GJs. For Niagara GJs, delphinidin-3-*O*-p-coumaroylglucoside bioaccessibility was significantly higher from 2013 compared to 2014 harvest GJ (24.3% ± 1.0% compared 9.5% ± 0.1%). Unlike non-anthocyanin phenolics, anthocyanin absolute bioaccessibility from each GJ were reflective of the trends observed in relative bioaccessibility. Notably, cyanidin-3,5-*O*-diglucoside had highest absolute bioaccessibility (13.4–16.2 nM). Absolute bioaccessibility was low for remaining anthocyanins ranging from 0.9 to 10.1 nM.

Following assessment of bioaccessibility, AQ fractions from final digesta of juices and sugar-matched controls were diluted with PBS (pH 5.5) containing 6 mM d7-glu and applied to the apical side of Caco-2 monolayers to determine intestinal glucose transport from phenolic rich GJ. Apical to basolateral transport of d7-glu was assessed over 60 min. Compared to digesta from phenolic-free sugar matched control beverages, all media containing GJ AQ digesta reduced d7-glu transport efficiency by 34.3% to 45.2% over 60 min (Table 5, Figure 2). These results were similar to those observed from GJ extracts described earlier (Figure 1) and suggest that activities observed in extract screen are preserved through digestion. 

To better understand the potential influence of macronutrients from a complex meal on the ability of GJ to influence carbohydrate digestion and glucose transport, Concord and SO_2_ Niagara GJ (2013 harvest) were co-formulated with a model test meal (1:1 and 1:2 (wt:wt) in starting material) consisting of a corn starch and milk protein rich model meal and digested in vitro. Co-digestion of starch-rich model meal with Concord and SO_2_ Niagara GJ at both high (1:1 GJ:model meal) and low (1:2 GJ:model meal) juice levels resulted in decreased glucose release (5.9% to 15.0% reduction) from starch digestion relative to phenolic-free sugar matched control (Table 6), suggesting bioaccessible phenolics from 100% GJ have ability to modulate glucose availability by decreasing the digestive release of glucose from starch in the small intestine. 

To confirm that bioaccessible GJ phenolics resulting from co-digestion of GJ with test meal maintain the ability to modulate d7-glu intestinal transport, AQ digesta fractions from co-digestion experiments were diluted with PBS containing a final concentration of 6 mM of d7-glu. Despite lower phenolic concentration in test media, distinct inhibition of glucose transport was observed (Table 5; Figure 3). Specifically, d7-glu transport was decreased by 4.8%–21.4%, with a significant (*p* < 0.05) decline in glucose transport observed for treatment with 50% Concord GJ inclusion in the model meal. 

## 4. Discussion

Clinical evidence exists to support the notion that certain phenolic-rich foods and beverages may modify glycemic parameters [22,23,41,42,43,44,45,46]. One hundred percent GJ is a particularly rich source of dietary phenolics but also naturally high in sugar (~36 g sugar per 240 mL serving) [9]. While it has been reported that fruit juices have similar glycemic responses to their corresponding whole fruits [10]), grape juice has also been shown to have a lower insulinemic response than corresponding grapes [21]. This may be related to the differential profile of grape juice compared to grapes and, thus, a better understanding of the interaction between GJ phenolics and the intrinsic sugar in these products is required. This study was designed to develop insight into the ability and mechanisms by which GJ phenolics may modulate starch digestion and absorption of glucose in the context of a juice matrix with and without a starch rich meal.

One mechanism that has been proposed for these effects is related to phenolic inhibition of starch digestion [47,48,49,50]. In the present study, GJ phenolic extracts (300 and 500 µM) demonstrated only modest inhibitory capacity for α-amylase and α-glucosidase (Table 3). Harvest year impacted α-amylase, but not α-glucosidase activity, with 2013 extracts generally having greater impact compared to 2014 extracts. This may be related to subtle differences in qualitative phenolic profiles from 2013 to 2014 harvest GJs as total levels appeared similar between harvest years. Phenolic rich GJ extracts had greater inhibitory activity toward α-glucosidase compared to α-amylase. Similar results were previously reported for wine grape tannins, pomace and skin extracts for inhibition of α-glucosidase (~20% to 85%), with little to no detectable impact on α-amylase activity [48,49]. Levels of phenolics required to achieve even modest inhibition were observed to be high (>300 µM). However, it is important to note that concentrations of phenolics in the gut lumen from a serving of phenolic rich food or beverage may in in fact be quite high and approach high µM to even mM levels as previously postulated [7,51]. Therefore, results here suggesting a modest ability of GJ phenolics to inhibit α-glucosidase at a 300 µM dose does support the hypothesis that consumption of GJ with starch rich foods may have relevance to starch digestion and liberation of glucose in the gut lumen.

A second mechanism by which phenolics may modulate glycemic response is through alteration of glucose intestinal transport [18,19,20]. In the present study, GJ extracts (10–100 µM total phenolics) reduced intestinal glucose and fructose transport by Caco-2 human intestinal cell monolayers compared to control. These findings are similar to those previously reported with plant-derived phenolic extracts [18,19,20] (Figure 1; Table S1). Overall, inhibition by GJ extracts was greater for d7-fru compared to d7-glu transport, with extracts from Niagara GJ generally exhibiting greater inhibitory activity relative to Concord GJ extracts. Since extracts were standardized for total phenolics, these results suggest that the qualitative phenolic profile of Niagara GJ, which is primarily composed of non-anthocyanin flavonoids and phenolic acids and minimal amounts of anthocyanins (Table 2), may be most critical to consider in selection of juices and therefore merits additional investigation as targeted modifiers of intestinal glucose transport. The mechanism behind this reduction of glucose transport may be related, in part, to the ability of GJ phenolics to inhibit expression of hexose transporters (GLUT2 and SGLT1) or through direct inhibition of these transporters. Alzaid et al. [20] demonstrated that GLUT2 and SGLT1 mRNA were significantly decreased compared to baseline by up to 85% and 70%, respectively, following treatment of cells with berry extract for over 12 h. However, although GLUT2 protein was significantly reduced compared to control by treatment of blueberry extract for 16 h, SGLT1 protein levels were not affected. In a preliminary experiment, expression of GLUT2 and SGLT1 mRNA was measured in Caco-2 monolayers exposed to 100% GJ phenolic extracts (SO_2_ Niagara and Concord 2013) for 4 and 24 h. Interestingly, GLUT2 mRNA was significantly (*p* < 0.05) decreased two-fold following treatment by Concord GJ phenolic extract, but no significant change in SGLT1 expression was observed (Figure S1). While these preliminary results are consistent with previous observations [18,19,20], they cannot fully explain the observed effects in the present study. Previous reports have also demonstrated that bioavailability of select polyphenols may be increased in the presence of carbohydrates [52,53,54] suggesting the potential for additional interrelated mechanism impacting the transport of both phenolics and carbohydrate. Transepithelial flux of grape juice phenolics was not simultaneously monitored in this study, and, as such, it is not possible to speculate to these mechanisms. Further investigations are therefore warranted to better delineate the extent to which phenolic inhibit of natural juice sugars may be related to changes in transporter expression or function, or alternative mechanisms. 

Finally, in order to better understand the extent to which these effects would be extendable to 100% GJ, Niagara and Concord GJ or matching phenolic free control were formulated with and without a starch rich model meal and digested in vitro. Differences observed in phenolic relative bioaccessibility from juice alone are suggestive of variations in grape phenolic components between harvest years (Table 2 and Table 4). On the other hand, SO_2_ treatment did not impact relative bioaccessibility of phenolics from Niagara GJ. These results logically suggest that starting concentration of phenolics in 100% GJ have a direct impact on concentration of phenolics available for interactions in the gut and ultimate stability and accessibility of phenolics in the small intestine. Following assessment of phenolic bioaccessibility, transport of glucose from AQ digesta of 100% GJ and sugar match controls by Caco-2 intestinal cells was assessed. All GJ AQ digesta reduced d7-glu transport efficiency compared to phenolic-free sugar matched control up to 45% over 60 min (Figure 2; Table 5). These results were similar to those observed from extract screening and suggest that reduced efficiency of intestinal glucose transport may be a factor to consider in assessing glycemic response from GJ relative to a phenolic free sugar sweetened beverage. Interestingly, this observation is consistent with the glycemic response of phenolic rich apple juice that has previously been shown to cause a modest reduction in glycemic response relative to sugar matched controls in healthy humans [22,41]. 

To build on these findings with the awareness that GJ is commonly consumed with meals, it is important to consider the consequences of co-consumed food on ability of GJ phenolics to modulate carbohydrate digestion and glucose transport. It is well known that phenolics interact non-covalently with protein and carbohydrate (reviewed by Bordenave et al. [55] and Jakobek et al. [56]). These interactions in the context of a co-consumed meal may result in changes to the activity of phenolics relative to endpoints critical to glycemic response, namely carbohydrate digestion and glucose transport. Therefore, digestion of carbohydrate from a starch and protein-rich test meal co-formulated with GJ compared to phenolic-free control was determined. Although the level of GJ phenolics in AQ digesta resulting from co-digestion of GJ with test meal was lower compared to level of phenolics from GJ extracts used in enzyme inhibition assays (Table 3 and Table 5), results for inhibition of starch digestion were in fact similar. Therefore, it appears bioaccessible phenolics in the context of a complex meal still have the ability to impact digestion of carbohydrates derived from the meal. The extent to which this may be due to the proximity of phenolics and macronutrients in the meal and specific interactions that may develop through preparation and/or digestion remains to be explored. 

Modifying glucose transport by GJ in the context of a digested meal was also determined. The effect of AQ digesta from GJ containing meals demonstrated only modest inhibitory effects (Figure 3) which reach significance only for 100% Concord GJ. While in contrast with extract screening that found Niagara phenolics to be more effective, this observation was not totally unexpected as the phenolic concentrations resulting from digestion of mixed meals were lower than extract and juice experiments, especially for Niagara (Table 5). Considering that in humans concentrations in the gut lumen may reach high μM to mM levels [12] from typical food doses and gut dilution, responses within a meal such as those observed with berries by Törrönen et al. [57] may be expected. While requiring additional clinical insights as to the direction and extent of this effect in vivo are required, current results, while in vitro, do reflect the modest but important changes in glycemic response observed in recent clinical trials involving phenolic-rich foods and beverages [22,23,41,42,43,44,45,46]. This is relevant considering that moderate post-prandial hyperglycemia blood glucose levels (148–199 mg/dL) have been shown to be indicative of the development of negative health effects including atherosclerosis and endothelial damage [58]. This range represents a ~6% increase compared to the recommended <140 mg/dL post-prandial (2 h) blood glucose target [59], suggesting that even subtle improvements to post-prandial blood glucose level may prevent development of negative health outcomes. Therefore, while subtle, the current observations that 100% GJ may impact both carbohydrate digestion and glucose transport both from juice and in the context of a model meal suggest that the benefits of 100% GJ may be extendable beyond the glucose derived from the juice and to the response of a full meal and, thus, have positive impacts on health. 

## 5. Conclusions 

Although high in natural sugar, 100% Concord or Niagara GJ remains a rich source of dietary phenolics that have been reported to modify glycemic parameters through alteration of carbohydrate digestion and glucose transport. Results of the current study are in general agreement with previous studies reporting the ability of phenolics to decrease α-glucosidase activity [48,49] and also indicate that 100% GJ phenolics have the ability to decrease glucose transport even following simulated oral gastric and small intestinal digestions. Further, results suggest that 100% GJ, when placed in the context of a meal, maintains the ability to decrease intestinal starch digestion and subsequent glucose transport, in a fashion consistent with promotion of healthy glucose homeostasis. Further clinical assessments of 100% GJ in the context of glycemic response are warranted to clarify the impact of both intrinsic fruit sugar in juice and the potential impact of fruit phenolics on glycemic response from a meal. 

## Figures and Tables

**Table 1:** Description of 100% grape juice samples assessed ^1^.

Grape Juice Description	Form of Juice	Sugar Content
Niagara, 2013 harvest	Reconstituted from concentrate	16.0° Brix
SO_2_ Niagara, 2013 harvest	Reconstituted from concentrate	16.0° Brix
Concord, 2013 harvest	Not from concentrate	16.5° Brix
Niagara, 2014 harvest	Reconstituted from concentrate	16.0° Brix
SO_2_ Niagara, 2014 harvest	Not from concentrate	13.3° Brix
Concord, 2014 harvest	Not from concentrate	15.9° Brix

^1^ All samples received from Welch’s Foods Inc.

**Table 2:** Content (µM) of individual non-anthocyanin phenolics and anthocyanins in three types of grape juices over two harvest years ^1,2,3,4^.

Phenolic Content (mg/100 mL)												
100% Juice: Grape, Harvest Year	Gallic Acid	Caffeic Acid	Caftaric Acid	Epicatechin	Quercetin 3-*O*-glucoside	Quercetin 3,4-*O*-diglucoside	Quercetin-3-*O*-glucuronide	Quercetin	Isorhamnetin	Piceid	Resveratrol	Sum
Niagara, 2013	5.0 ± 0.4 ^b^	3.4 ± 0.1 ^c^	4.2 ± 0.1 ^e^	NC	NC	2.2 ± 0.2 ^c^	NC	16.8 ± 0.2 ^d^	4.9 ± 0.1 ^b^	0.8 ± 0.01 ^f^	7.1 ± 0.2 ^d^	44.5 ± 1.2 ^e^
SO_2_ Niagara, 2013	1.9 ± 0.04 ^d^	5.2 ± 0.5 ^b,c^	9.6 ± 0.2 ^d^	1.5 ± 0.1 ^c^	NC	2.0 ± 0.2 ^c^	NC	19.8 ± 1.1 ^c^	6.2 ± 0.4 ^b^	5.3 ± 0.3 ^a^	13.3 ± 0.7 ^a^	64.8 ± 3.2 ^d^
Concord, 2013	8.1 ± 0.4 ^a^	11.1 ± 0.2 ^a^	20.8 ± 0.7 ^b^	7.9 ± 0.7 ^b^	5.9 ± 0.4 ^b^	4.2 ± 0.2 ^a^	4.9 ± 0.3 ^a^	30.0 ± 0.5 ^b^	12.6 ± 0.4 ^a^	2.2 ± 0.02 ^d^	3.5 ± 0.1 ^f^	111.1 ± 3.8 ^b^
Niagara, 2014	4.0 ± 0.2 ^b,c^	4.2 ± 0.2 ^c^	2.9 ± 0.1 ^e^	2.4 ± 0.3 ^c^	NC	2.5 ± 0.2 ^c^	NC	20.6 ± 0.1 ^c^	5.0 ± 0.1 ^b^	1.4 ± 0.03 ^e^	10.3 ± 0.1 ^c^	53.4 ± 1.3 ^e^
SO_2_ Niagara, 2014	2.9 ± 0.6 ^c,d^	7.6 ± 1.7 ^b^	16.9 ± 0.9 ^c^	11.6 ± 0.9 ^a^	NC	3.3 ± 0.4 ^b^	NC	31.1 ± 0.6 ^b^	10.3 ± 1.3 ^a^	4.9 ± 0.06 ^b^	11.7 ± 0.01 ^b^	100.4 ± 6.7 ^c^
Concord, 2014	8.9 ± 0.4 ^a^	12.8 ± 0.6 ^a^	25.1 ± 0.7 ^a^	12.6 ± 1.2 ^a^	7.8 ± 0.5 ^a^	4.3 ± 0.3 ^a^	3.8 ± 0.2 ^b^	34.3 ± 0.3 ^a^	13.1 ± 0.5 ^a^	4.3 ± 0.2 ^c^	5.1 ± 0.2 ^e^	131.9 ± 4.9 ^a^
**Anthocyanin Content (ng/100 mL)**												
**100% Juice: Grape, Harvest Year**	**Cyanidin-3,5-*O*-diglucoside**		**Cyanidin-3-*O*-glucoside**		**Cyanidin-3-*O*-acetyl glucoside**	**Peonidin-3,5-*O*-diglucoside**	**Peonidin-3-*O*-glucoside**	**Peonidin-3-*O*-acetyl glucoside**	**Delphinidin-3-*O*-glucoside**		**Delphinidin-3-*O*-acetyl glucoside**	
Concord, 2013	623.5 ± 38.7 ^a^		74.8 ± 7.9 ^b^		87.5 ± 15.8 ^a^	144.7 ± 13.5 ^a^	13.6 ± 2.6 ^b^	10.7 ± 2.4 ^a^	620.5 ± 45.0 ^b^		113.2 ± 13.0 ^b^	
Concord, 2014	710.0 ± 60.5 ^a^		106.9 ± 13.4 ^a^		40.8 ± 17.4 ^a^	150.4 ± 11.5 ^a^	19.2 ± 2.0 ^a^	15.9 ± 3.3 ^a^	877.4 ± 66.0 ^a^		210.3 ± 27.7 ^a^	
**Anthocyanin Content (ng/100 mL)**												
**100% Juice: Grape, Harvest Year**	**Delphinidin-3-*O*-p-coumaroyl-5-*O*-diglucoside**		**Delphinidin-3-*O*-p-coumaroyl glucoside**		**Petunidin-3-*O*-glucoside**	**Petunidin-3-*O*-acetyl glucoside**	**Petunidin-3-*O*-p-coumaroyl-5-O-diglucoside**	**Malvidin-3-*O*-glucoside**	**Malvidin-3-*O*-acetyl glucoside**		**Sum**	
Concord, 2013	122.3 ± 20.6 ^b^		199.8 ± 6.0 ^a^		191.1 ± 17.2 ^a^	35.0 ± 3.5 ^b^	35.3 ± 7.4 ^a^	133.4 ± 8.0 ^b^	22.3 ± 3.3 ^b^		2427.5 ± 205.0 ^b^	
Concord, 2014	212.1 ± 40.8 ^a^		211.7 ± 9.1 ^a^		210.1 ± 20.2 ^a^	61.9 ± 10.1 ^a^	58.2 ± 11.7 ^a^	168.4 ± 14.5 ^a^	38.8 ± 6.4 ^a^		3092.0 ± 314.6 ^a^	

^1^ Values represent mean ± standard error of mean from a triplicate analysis; ^2^ NC = Non Detected; ^3^ Presence of different letter (a, b) between values indicates significant differences in concentration of phenolic class between grape juices (*p* < 0.05); ^4^ Cyanidin-3-*O*-p-coumaroyl-5-*O*-diglucoside and delphinidin-3-*O*-p-coumaroyl glucoside were present in Niagara juices at 0.9 ± 0.01 to 1.9 ± 0.1 and 3.7 ± 0.1 to 4.1 ± 0.1 µM, respectively.

**Table 3:** Inhibition (%) of α-amylase and glucosidase activity by grape juice phenolic extracts.

Inhibitor	Inhibitor Concentration (μM GAE) ^1^	Percent (%) Inhibition	
		α-Amylase	α-Glucosidase
Negative Control	0	0	0
Acarbose (Positive Control)	500	103.2 ± 5.1	92.4 ± 1.2
	300	102.0 ± 6.1	88.9 ± 0.9
	5	30.5 ± 2.9	6.2 ± 0.8
	3	17.6 ± 5.1	2.3 ± 0.5
Niagara, 2013	500	7.9 ± 4.5 ^a,b,^*	10.0 ± 4.2 ^a,b,^*
	300	4.5 ± 2.2 ^b,c,^*	6.6 ± 2.9 ^a,b,^*
SO_2_ Niagara, 2013	500	9.4 ± 3.3 ^a,^*	11.5 ± 3.1 ^a,^*
	300	−3.9 ± 2.0 ^f^	7.1 ± 2.6 ^a,b,^*
Concord 2013	500	8.7 ± 4.3 ^a,^*	9.2 ± 0.8 ^a,b,^*
	300	−3.4 ± 2.0 ^f^	5.4 ± 2.1 ^b,^*
Niagara, 2014	500	0.7 ± 1.7 ^d,e^	6.2 ± 3.4 ^a,b,^*
	300	−1.9 ± 2.6 ^e,f^	3.8 ± 2.0 ^b^
SO_2_ Niagara, 2014	500	9.2 ± 3.9 ^a,^*	7.1 ± 2.7 ^a,b,^*
	300	0.5 ± 1.0 ^d,e^	4.2 ± 2.1 ^b^
Concord 2014	500	3.4 ± 1.4 ^c,d,^*	9.3 ± 3.2 ^a,b,^*
	300	−1.2 ± 2.1 ^e,f^	4.9 ± 1.2 ^b^

Experiments represent average of *n* = 3 replicates; Preliminary dose finding experiments conducted with range of 10–1000 μM of phenolic extracts; Presence of different letter between values indicates significant differences in percentage inhibition between GJ extracts (*p* < 0.05); * indicates significant differences in percent inhibition by inhibitor compared to negative control (*p* < 0.05); ^1^ Total phenolics in digesta determined using Folin-Ciocalteu Assay and expressed as gallic acid equivalents (GAE); HPAEC-ECD and inhibition of α-glucosidase by glucose oxidase-peroxidase assay.

**Table 4:** Relative (%) and absolute (µM or nM) bioaccessibility of non-anthocyanin phenolics and anthocyanins for three types of grape juices over two harvest years ^1,2,3,4,5^.

Non-Anthocyanin Phenolic Relative Bioaccessibility (%)									
100% Juice: Grape, Harvest Year	Gallic Acid	Caffeic Acid	Caftaric Acid	Epicatechin	Quercetin 3,4-diglucoside	Quercetin	Isorhamnetin	Piceid	Resveratrol
Niagara, 2013	32.0 ± 9.2 ^a^	24.8 ± 2.6 ^b^	39.5 ± 0.6 ^a,b^	NC ^e^	16.9 ± 6.8 ^a^	8.4 ± 0.8 ^a^	20.9 ± 0.8 ^a^	22.1 ± 1.2 ^a^	18.7 ± 1.9 ^b^
SO_2_ Niagara, 2013	31.7 ± 4.4 ^a^	36.1 ± 4.2 ^a,b^	32.7 ± 1.7 ^b^	27.4 ± 3.0 ^a^	21.7 ± 8.5 ^a^	7.6 ± 0.5 ^a^	18.5 ± 1.9 ^a^	10.3 ± 0.3 ^c,d^	17.5 ± 1.6 ^b^
Concord, 2013	29.6 ± 6.6 ^a^	29.1 ± 3.6 ^b^	32.2 ± 1.6 ^b^	18.5 ± 1.4 ^c^	15.7 ± 7.4 ^a^	2.6 ± 0.5 ^b^	7.6 ± 0.4 ^b^	14.8 ± 2.0 ^b^	26.6 ± 1.7 ^a^
Niagara, 2014	29.7 ± 5.8 ^a^	27.5 ± 2.0 ^b^	56.0 ± 15.0 ^a^	11.8 ± 2.0 ^d^	13.8 ± 4.6 ^a^	2.1 ± 0.2 ^b^	7.6 ± 0.2 ^b^	12.6 ± 0.1 ^b,c^	14.6 ± 1.4 ^b^
SO_2_ Niagara, 2014	22.0 ± 1.5 ^a^	44.7 ± 8.0 ^a^	31.6 ± 3.2 ^b^	24.3 ± 2.1 ^a,b^	16.7 ± 8.8 ^a^	1.7 ± 0.3 ^b^	4.1 ± 0.4 ^c^	8.9 ± 1.3 ^c,d^	16.8 ± 2.3 ^b^
Concord, 2014	29.2 ± 2.5 ^a^	28.7 ± 5.7 ^b^	30.3 ± 5.2 ^b^	21.3 ± 1.3 ^b,c^	12.2 ± 5.0 ^a^	1.8 ± 0.5 ^b^	3.3 ± 0.3 ^c^	8.1 ± 1.2 ^d^	17.6 ± 3.8 ^b^
**Anthocyanin Relative Bioaccessibility (%)**									
**100% Juice: Grape, Harvest Year**	**Cyanidin-3,5-*O*-diglucoside**	**Peonidin-3,5-*O*-dglucoside**	**Delphinidin-3-*O*-glucoside**	**Delphinidin-3-*O*-acetyl glucoside**		**Delphinidin-3-*O*-p-coumaroyl-glucoside**		**Petunidin-3-*O*-glucoside**	**Malvidin-3-*O*-glucoside**
Concord, 2013	18.0 ± 0.9 ^a^	25.7 ± 1.5 ^a^	12.0 ± 1.1 ^a^	6.3 ± 1.8 ^a^		10.1 ± 0.8 ^a^		23.0 ± 1.9 ^a^	37.2 ± 1.7 ^a^
Concord, 2014	13.1 ± 1.2 ^b^	18.7 ± 1.1 ^b^	8.1 ± 1.3 ^b^	3.7 ± 0.9 ^b^		6.3 ± 0.6 ^b^		19.9 ± 2.9 ^a^	30.0 ± 4.8 ^b^
**Non-Anthocyanin Phenolic Absolute Bioaccessibility (µM)**									
**100% Juice: Grape, Harvest Year**	**Gallic Acid**	**Caffeic Acid**	**Caftaric Acid**	**Epicatechin**	**Quercetin 3,4-diglucoside**	**Quercetin**	**Isorhamnetin**	**Piceid**	**Resveratrol**
Niagara, 2013	91.0 ± 20.0 ^b^	47.5 ± 6.2 ^b^	53.4 ± 0.6 ^e^	NC	8.2 ± 3.4 ^a^	46.5 ± 3.7 ^a^	21.2 ± 0.7 ^b^	8.2 ± 0.4 ^c^	57.8 ± 4.6 ^c,d^
SO_2_ Niagara, 2013	36.3 ± 5.6 ^c^	103.6 ± 8.8 ^b^	100.9 ± 5.2 ^d^	13.7 ± 1.3 ^c^	9.2 ± 3.7 ^a^	50.3 ± 5.6 ^a^	23.7 ± 1.1 ^a^	24.2 ± 1.6 ^a^	102.7 ± 14.0 ^a^
Concord, 2013	139.0 ± 25.9 ^a^	179.1 ± 22.3 ^a^	214.3 ± 8.8 ^b^	50.7 ± 7.1 ^b^	14.3 ± 6.9 ^a^	25.9 ± 4.7 ^b^	19.9 ± 0.7 ^b^	14.1 ± 1.9 ^b^	40.4 ± 1.8 ^c,d^
Niagara, 2014	68.7 ± 10.6 ^b,c^	64.8 ± 6.3 ^b^	53.3 ± 15.2 ^e^	9.5 ± 0.9 ^c^	7.8 ± 3.4 ^a^	14.0 ± 1.2 ^b^	8.0 ± 0.1 ^c^	7.8 ± 0.2 ^c^	66.1 ± 6.9 ^b,c^
SO_2_ Niagara, 2014	37.0 ± 6.2 ^c^	177.6 ± 37.1 ^a^	169.2 ± 7.2 ^c^	96.8 ± 10.5 ^a^	10.5 ± 4.4 ^a^	17.8 ± 2.6 ^b^	8.7 ± 0.2 ^c^	19.2 ± 2.6 ^a,b^	86.1 ± 11.6 ^a,b^
Concord, 2014	152.4 ± 14.2 ^a^	202.5 ± 37.4 ^a^	241.1 ± 35.8 ^a^	91.5 ± 3.7 ^a^	11.5 ± 4.6 ^a^	20.3 ± 5.1 ^b^	8.9 ± 0.5 ^c^	15.3 ± 2.2 ^b^	38.6 ± 7.7 ^d^
**Anthocyanin Absolute Bioaccessibility (nM)**									
**100% Juice: Grape, Harvest Year**	**Cyanidin-3,5-*O*-diglucoside**	**Peonidin-3,5-*O*-dglucoside**	**Delphinidin-3-*O*-glucoside**		**Delphinidin-3-*O*-acetyl glucoside**		**Delphinidin-3-*O*-p-coumaroyl-glucoside**	**Petunidin-3-*O*-glucoside**	**Malvidin-3-*O*-glucoside**
Concord, 2013	16.2 ± 1.0 ^a^	5.1 ± 0.4 ^a^	10.1 ± 0.4 ^a^		0.9 ± 0.2 ^a^		2.8 ± 0.2 ^a^	5.7 ± 0.2 ^a^	6.2 ± 0.3 ^a^
Concord, 2014	13.4 ± 1.5 ^b^	3.9 ± 0.2 ^b^	9.8 ± 1.7 ^a^		1.0 ± 0.2 ^a^		1.8 ± 0.3 ^b^	5.6 ± 1.4 ^a^	6.4 ± 1.4 ^a^

^1^ Values represent mean ± standard error of mean from a triplicate analysis; ^2^ NC = Non Detected; ^3^Presence of different letter between values indicates significant differences in concentration of phenolic class between grape juices (*p* < 0.05); ^4^ Quercetin-3-*O*-glucoside bioaccessibility from Concord grape juices was between 7.2% ± 2.7% to 8.1% ± 3.5%. Delphinidin-3-*O*-p-coumaroyl glucoside bioaccessibility from Niagara juices and Concord juices was between 8.4% ± 0.5% to 24.3% ± 1.0% and 6.3% ± 0.6% to 10.1% ± 0.8%, respectively; ^5^ Phenolics in GJs and digesta determined using LC-MS.

**Table 5:** Glucose transport by Caco-2 small intestinal epithelial cells co-treated with d7-Glu (6 mM) and aqueous digesta (AQ) or matched phenolic-free control ^1,2,3,4^.

Treatment	Phenolic Concentration (μM) ^5^	Percent d7-Glu Transport ^6^	Percent (%) d7-Glu Transported over 60 min Relative to Control ^7^
Without Model Test Meal			
Control (24 mM glucose/fructose)	0	2.4 ± 0.1 ^a^	100 ^a^
Niagara, 2013 AQ	27.1	1.3 ± 0.1 ^b^	54.8 ± 5.4 ^b^
SO_2_ Niagara, 2013 AQ	37.8	1.4 ± 0.3 ^b^	58.6 ± 13.0 ^b^
Concord, 2013 AQ	56.3	1.4 ± 0.2 ^b^	59.2 ± 7.3 ^b^
Niagara, 2014 AQ	20.8	1.4 ± 0.3 ^b^	58.2 ± 10.4 ^b^
SO_2_ Niagara, 2014 AQ	39.4	1.4 ± 0.2 ^b^	60.1 ± 9.0 ^b^
Concord 2014 AQ	53.7	1.6 ± 0.2 ^b^	65.7 ± 6.5 ^b^
With Model Test Meal			
1:1 Control (12 mM glu/fru)	0	1.5 ± 0.1 ^a^	100 ^a^
1:2 Control (6 mM glu/fru)	0	1.3 ± 0.1 ^a^	100 ^a^
1:1 Concord 2013	16.4	1.1 ± 0.2 ^b^	78.6 ± 7.7 ^b^
1:1 SO_2_ Niagara 2013	7.1	1.3 ± 0.2 ^a,b^	90.6 ± 7.6 ^a,b^
1:2 Concord, 2013	10.1	1.3 ± 0.1 ^a^	89.5 ± 9.5 ^a,b^
1:2 SO_2_ Niagara 2013	4.6	1.4 ± 0.1 ^a^	95.2 ± 10.2 ^a^

^1^ Treatments included aqueous digesta (AQ) diluted 2:7 prior to introduction to apical compartment of three-compartment Caco-2 cell model; ^2^ d7-Glucose (6 mM) was used as a marker for glucose transport; Diluted Concord, Niagara, and blank digesta AQ contained 15, 12, and 12 mM glucose, respectively and 12, 17, and 12 mM fructose, respectively; ^3^ Data represent an average of *n* = 4 wells per experiment; ^4^ Presence of different letter between values indicates significant differences in glucose transport between treatment and control, within experiment comparing AQ digesta from different juices or experiment comparing AQ digesta with and without model test meal (*p* < 0.05); ^5^ Total phenolics and sugars in digesta determined using LC-MS; ^6^ Percent of d7-glucose transported from apical media to basolateral compartment; ^7^ Amount of d7-glucose transported basolaterally over 60 min relative to daily control matched for glucose/fructose and d7-glucose.

**Table 6:** Percentage decrease in release of glucose from starch-rich model meal co-digested with grape juice compared with a sugar-matched control ^1^.

Formulation ^2^	Concentration of Phenolics in Aqueous Digesta (AQ) Fraction Following Digestion (µM) ^3^	Percent Decrease in Glucose Release from Corn Starch by GJ Phenolics Compared to Phenolic-Free Control ^4^
1:1 Concord:Model meal	73.7	15.0
1:2 Concord: Model meal	35.4	5.9
1:1 SO_2_ Niagara: Model meal	25.9	12.1
1:2 SO_2_ Niagara: Model meal	17.2	6.6

^1^ Experiments represent average of *n* = 3 replicates; ^2^ High and low 100% Concord 2013 GJ contained 473 and 236 µmol/240 mL serving total phenolics, respectively; High and low Niagara 2013 GJ contained 289 and 144 µmol/240 mL serving total phenolics, respectively; Concord and Niagara 2013 juices contained 447 and 596 mM fructose, respectively, and 531 and 427 mM glucose, respectively (determined using LC-MS); ^3^ Total phenolics and sugars in SM and AQ digesta determined using LC-MS; ^4^ Phenolic-free control was distilled water with matching glucose and fructose content.

## Supplementary Materials

The following are available online at http://www.mdpi.com/2072-6643/8/7/414/s1, Table S1: Glucose transport by Caco-2 small intestinal epithelial cells co-treated with sugar solution (Glu and Fru (9 mM each) and d7-Glu and d7-Fru (3 mM each) and phenolic extract from grape juice or phenolic-free control matched for sugar content, Table S2: Relative (%) bioaccessibility of marker phenolics and anthocyanins in Concord, 2013 grape juice co-digested with a protein containing corn starch gel (model meal), Table S3: Forward and reverse PCR primer sequences utilized for gene expression analysis by PCR, Figure S1: Impact grape juice phenolic extract (50 μM) treatment (4 or 24 h) has on Caco-2 expression of SGLT1 or GLUT2 compared to phenolic-free control. Data is represented as protein expression compared to control β-actin. Data represent mean ± SEM for *n* = 4 replicate wells. Presence of different letter between values indicates significant differences in protein expression between treatments within each time point (*p* < 0.05).

## Abbreviations

The following abbreviations are used in this manuscript:

GJ	Grape juice
d7-Glu	Deuterated glucose
d7-Glu	Deuterated fructose
LDL	low density lipoprotein
GLUT2	glucose transporter 2
SGLT1	sodium-dependent glucose transporter 1
DMEM	Dulbecco’s Modified Eagles Medium
NEAA	non-essential amino acids
Pen/strep	penicillin/streptomycin
HEPES	4-(2-hydroxylethyl)-1-piperazineethanes
FBS	fetal bovine serum
FFA	bovine serum albumin (free fatty acid free)
SPE	solid phase extraction
LC-MS	liquid chromatography-mass spectrometry
DMSO	dimethylsulfoxide
PBS	phosphate buffered saline
SM	starting material
AQ	aqueous (intestinal digesta)
NFDM	non-fat dry milk
HPAEC-ECD	high pressure anion exchange chromatography-electrochemical detector
PCR	polymerase chain reaction

## Appendix A

### Appendix A.1. Modulation of Caco-2 Glucose/Fructose Transporter Gene Expression by GJ Phenolic Extracts

Cells and experimental conditions for gene expression experiment were maintained and executed according to glucose transport experiments, with minor modifications. Test media was prepared by solubilizing glucose and fructose (12 mM each) and GJ phenolic extracts in PBS pH 5.5 (delivering 50 µM total phenolics). Test media was applied to the apical surface of cell monolayers and cells were incubated for 4 or 24 h. Cells were collected with RNAlater (Sigma-Aldrich) and frozen until analysis. Treatments were replicated in quadruplicate.

### Appendix A.2. Characterization of mRNA Expression and Anthocyanin-Rich Fractions by LC-MS

Total RNA was isolated from the cultured cells using TRIzol^®^ (Invitrogen™ Life Technologies, Paisley, UK) according to the manufacturer’s instructions. RNA concentration was measured using wavelength of 260 and 280 using a NanoDrop 2000 UV-Vis Spectrophotometer (Thermo Scientific., Wilmington, DC, USA) Following first strand cDNA synthesis, expression levels of glucose transporter mRNA and GAPDH mRNA (used as a housekeeping gene) were analysed by real-time quantitative PCR using an ABI Prism 7700HT Sequence Detection System and a Power SYBR^®^ Green PCR master mix kit (Applied Biosystems™, Cheshire, UK). The primer sequences used for each gene are given in Table S3. cDNA was then synthesized from 150 ng RNA using M-MuLV reverse transcriptase (BioLabs, Ipswich, MA, USA). Real-time q-RT-PCR was performed using a CFX Connect Real-Time PCR Detection System (BioRad, Hercules, CA, USA). The expressions of SGLT1 and GLUT2 were evaluated and the expression of housekeeping gene β-actin was assessed. The primer sequences used for each gene are given in Table S3.
