# Supplementary material for: Concord and Niagara Grape Juice and Their Phenolics Modify Intestinal Glucose Transport in a Coupled in Vitro Digestion/Caco-2 Human Intestinal Model
Source: Nutrients. 2016 Jul 5;8(7):414. doi: 10.3390/nu8070414 (PMC4963890; doi:10.3390/nu8070414)
Supplement: Supplementary file 1 [file nutrients-08-00414-s001.docx]

Supplementary Materials: Concord and Niagara Grape Juice and Their Phenolics Modify Intestinal Glucose Transport in a Coupled in Vitro Digestion/Caco-2 Human Intestinal Model

Sydney Moser, Jongbin Lim, Mohammad Chegeni, JoLynne D. Wightman, Bruce R. Hamaker and Mario G. Ferruzzi

**Table S1.** Glucose transport by Caco-2 small intestinal epithelial cells co-treated with sugar solution (Glu and Fru (9 mM each) and d7-Glu and d7-Fru (3 mM each) and phenolic extract from grape juice or phenolic-free control matched for sugar content ^1,2,3^.

| **Treatment** | **Phenolic Concentration (μM GAE) ^4^** | **Percent d7-Fru Transport ^5^** | **Percent d7-Glu Transport ^5^** | **Percent (%) d7-Fru Transported over 60 min Relative to Control ^6^** | **Percent (%) d7-Glu Transported over 60 min Relative to Control ^6^** |
| --- | --- | --- | --- | --- | --- |
| Control (24 mM Glucose/Fructose) | 0 | 2.8 ± 0.2 ^a^ | 3.4 ± 0.3 ^a^ | 100 ^a^ | 100 ^a^ |
| Niagara, 2013 extract | 10 | 2.5 ± 0.2 ^a,b^ | 2.8 ± 0.2 ^a,b^ | 89.5 ± 5.8 ^a,b^ | 82.3 ± 5.9 ^a,b^ |
|  | 50 | 2.0 ± 0.1 ^c,^* | 2.4 ± 0.04 ^b,^* | 72.0 ± 2.0 ^c,^* | 71.5 ± 1.0 ^b,c,^* |
|  | 100 | 2.1 ± 0.1 ^b,c,^* | 2.3 ± 0.1 ^b,^* | 73.9 ± 4.0 ^b,c,^* | 66.5 ± 4.0 ^c,^* |
| SO_2_ Niagara, 2013 extract | 10 | 2.5 ± 0.1 ^a,b^ | 3.0 ± 0.2 ^a,b^ | 88.3 ± 4.6 ^a^ | 88.3 ± 3.0 ^a^ |
|  | 50 | 1.9 ± 0.1 ^b,^* | 2.8 ± 0.1 ^b,c,^* | 68.3 ± 3.0 ^b,^* | 83.0 ± 2.7 ^a,b^ |
|  | 100 | 2.0 ± 0.1 ^a,b,^* | 2.4 ± 0.1 ^c,^* | 70.5 ± 4.8 ^b,^* | 70.3 ± 3.9 ^b,^* |
| Concord 2013 extract | 10 | 2.1 ± 0.2 ^b,^* | 2.9 ± 0.2 ^a^ | 74.8 ± 6.1 ^b,^* | 84.6 ± 4.7 ^a^ |
|  | 50 | 2.2 ± 0.1 ^b,^* | 3.1 ± 0.2 ^a^ | 77.9 ± 3.9 ^b,^* | 89.2 ± 5.8 ^a^ |
|  | 100 | 2.0 ± 0.2 ^b,^* | 2.8 ± 0.2 ^a^ | 69.1 ± 6.4 ^b,^* | 82.3 ± 6.0 ^a^ |
| Niagara, 2014 extract | 10 | 2.3 ± 0.1 ^a,b^ | 3.0 ± 0.1 ^a^ | 85.55 ± 5.4 ^a,b^ | 95.3 ± 2.6 ^a^ |
|  | 50 | 2.1 ± 0.2 ^a,b^ | 3.0 ± 0.2 ^a^ | 78.9 ± 6.3 ^a,b^ | 95.2 ± 6.8 ^a^ |
|  | 100 | 1.6 ± 0.3 ^b,^* | 2.4 ± 0.4 ^a^ | 58.7 ± 12.4 ^b,^* | 77.8 ± 12.4 ^a^ |
| SO_2_ Niagara, 2014 extract | 10 | 2.3 ± 0.1 ^a,b^ | 2.8 ± 0.1 ^a^ | 87.1 ± 2.7 ^a,b^ | 89.7 ± 1.6 ^a,b^ |
|  | 50 | 2.0 ± 0.2 ^b,c,^* | 2.7 ± 0.2 ^a^ | 76.7 ± 7.3 ^b,c,^* | 86.0 ± 6.0 ^a,b^ |
|  | 100 | 1.6 ± 0.1 ^c,^* | 2.0 ± 0.3 ^b,^* | 59.6 ± 3.6 ^c,^* | 64.3 ± 9.1 ^b,^* |
| Concord, 2014 extract | 10 | 2.4 ± 0.1 ^a^ | 2.9 ± 0.2 ^a^ | 89.1 ± 5.4 ^a^ | 94.2 ± 5.9 ^a^ |
|  | 50 | 2.2 ± 0.1 ^a^ | 2.8 ± 0.1 ^a^ | 81.7 ± 5.6 ^a^ | 89.0 ± 2.4 ^a^ |
|  | 100 | 2.2 ± 0.2 ^a^ | 2.7 ± 0.2 ^a^ | 81.9 ± 8.4 ^a^ | 85.8 ± 7.0 ^a^ |

^1^ d7-Glucose and d7-fructose (3 mM) were used as markers for glucose transport; ^2^ Data represent an average of *n* = 4 wells per experiment; ^3^ Presence of different letter between values indicates significant differences in glucose transport within treatment and compared to control (*p* < 0.05); ^4^ Total phenolics in digesta determined using Folin-Ciocalteu assay, reported as gallic acid equivalents (GAE); ^5^ Percent of d7-fructose/glucose transported from apical media to basolateral compartment; ^6^ Amount of d7-fructose/glucose transported basolaterally over 60 min relative to daily control matched for glucose/fructose (9 mM each) and d7-glucose/d7-fructose (3 mM each); * Asterisks indicate transport is significantly decreased compared to phenolic-free control.

**Table S2.** Relative (%) bioaccessibility of marker phenolics and anthocyanins in Concord, 2013 grape juice co-digested with a protein containing corn starch gel (pudding) ^1,2^.

| **Phenolic Bioaccessibility (%)** | | | | | | | | | | | | | | |
| --- | --- | --- | --- | --- | --- | --- | --- | --- | --- | --- | --- | --- | --- | --- |
|  | **Gallic Acid** | **Caffeic Acid** | | **Caftaric Acid** | **Epicatechin** | | **Quercetin 3-*O*-Glucoside** | | **Quercetin-3,4-Diglucoside** | **Quercetin** | | **Isorhamnetin** | **Piceid** | **Resveratrol** |
| 1:2 Concord:Pudding | 41.8 ± 2.3 ^a^ | 63.1 ± 6.3 ^a^ | | 40.3 ± 3.1 ^a^ | 8.8 ± 1.3 ^b^ | | 4.6 ± 0.9 ^a^ | | 2.2 ± 0.1 ^b^ | 4.0 ± 0.1 ^b^ | | 5.7 ± 0.4 ^a^ | 0.8 ± 0.1 ^c^ | 1.5 ± 0.2 ^c^ |
| 1:2 Concord:Pudding | 19.5 ± 1.4 ^c^ | 31.1 ± 2.9 ^c^ | | 14.7 ± 1.7 ^b^ | 4.1 ± 0.2 ^c^ | | 1.3 ± 0.1 ^b^ | | 0.4 ± 0.1 ^d^ | 2.0 ± 0.2 ^d^ | | 5.1 ± 1.4 ^a^ | 0.5 ± 0.05 ^d^ | 0.8 ± 0.1 ^d^ |
| 1:2 Concord:Water | 33.8 ± 1.2 ^b^ | 47.1 ± 1.4 ^b^ | | 15.5 ± 0.5 ^b^ | 17.1 ± 0.3 ^a^ | | 5.1 ± 0.2 ^a^ | | 5.0 ± 0.02 ^a^ | 6.7 ± 0.1 ^a^ | | 2.5 ± 0.1 ^b^ | 1.9 ± 0.04 ^a^ | 3.5 ± 0.1 ^a^ |
| 1:2 Concord:Water | 16.0 ± 0.6 ^c^ | 21.7 ± 0.9 ^c^ | | 5.6 ± 0.4 ^c^ | 7.5 ± 0.2 ^b^ | | 1.3 ± 0.02 ^b^ | | 1.5 ± 0.1 ^c^ | 4.3 ± 0.2 ^c^ | | 1.0 ± 0.02 ^b^ | 1.2 ± 0.04 ^b^ | 1.9 ± 0.2 ^b^ |
| **Anthocyanin Bioaccessibility (%)** | | | | | | | | | | | | | | |
|  | **Cyanidin-3,5-*O*-Diglucoside** | | **Peonidin-3,5-*O*-Diglucoside** | | | **Delphinidin-3-*O*-Glucoside** | | **Delphinidin-3-*O*-p-Coumaroylglucoside** | | | **Petunidin-3-*O*-Glucoside** | | **Malvidin-3-*O*-Glucoside** | |
| 1:2 Concord:Pudding | 17.9 ± 1.4 ^b^ | | 21.2 ± 1.7 ^b^ | | | 20.3 ± 1.2 ^b^ | | 19.1 ± 4.2 ^a^ | | | 19.7 ± 4.6 ^b^ | | 31.3 ± 2.4 ^b^ | |
| 1:3 Concord:Pudding | 11.4 ± 1.1 ^c^ | | 10.7 ± 1.1 ^c^ | | | 4.9 ± 0.3 ^d^ | | 11.4 ± 1.7 ^a,b^ | | | 12.8 ± 1.3 ^b^ | | 16.4 ± 1.2 ^c^ | |
| 1:2 Concord:Water | 30.1 ± 1.8 ^a^ | | 39.6 ± 0.5 ^a^ | | | 27.7 ± 1.6 ^a^ | | 15.4 ± 0.9 ^a,b^ | | | 39.5 ± 1.4 ^a^ | | 46.1 ± 3.1 ^a^ | |
| 1:2 Concord:Water | 14.9 ± 0.4 ^b,c^ | | 22.6 ± 0.1 ^b^ | | | 12.4 ± 0.8 ^c^ | | 6.7 ± 0.3 ^b^ | | | 18.5 ± 0.6 ^b^ | | 23.8 ± 0.2 ^b,c^ | |

^1^ Values represent mean ± standard error of mean from a triplicate analysis; ^2^ Presence of different letter between values indicates significant differences in concentration of phenolic class between grape juices (*p* < 0.05).

**Table S3.** Forward and reverse PCR primer sequences utilized for gene expression analysis by PCR ^1^.

| **mRNA Target** | **Oligonucleotides (5′ → 3′)** |
| --- | --- |
| SGLT1 | F: TGGCAATCACTGCCCTTTA |
|  | R: TGCAAGGTGTCCGTGTAAAT |
| GLUT2 | F: GTCCAGAAAGCCCCAGATACC |
|  | R: GTGACATCCTCAGTTCCTCTTAG |
| Β-actin | F: TCCTATGTGGGTGACGAGGC |
|  | R: CATGGCTGGGGTGTTGAAGG |

^1^ F indicates forward primer, R indicates reverse primer.

**Figure S1.** Impact grape juice phenolic extract (50 μM) treatment (4 or 24 h) has on Caco-2 expression of SGLT1 or GLUT2 compared to phenolic-free control. Data is represented as protein expression compared to control β-actin. Data represent mean ± SEM for *n* = 4 replicate wells. Presence of different letter between values indicates significant differences in protein expression between treatments within each time point (*p* < 0.05).
